# Project Whiteboard: A Quality Improvement Project Enhancing Patient Flow in an Acute Mental Health Setting

**DOI:** 10.1192/bjo.2024.378

**Published:** 2024-08-01

**Authors:** Ashu Handa, Terry Harper, Brandon Wong, Kisa Abbas, Olanrewaju Odeyemi

**Affiliations:** CNWL, London, United Kingdom

## Abstract

**Aims:**

We are a 17 bedded acute mental health ward in a busy inner-city hospital. A handover of all patients, with the multi-disciplinary team, takes place every morning (Whiteboard round). The clinical team felt that the information provided during this meeting needed a review, to ensure relevant patient information is being disseminated, and right clinical decisions are being made in a timely manner.

The team decided to focus on improving links with Community Mental Health Hubs (CMHH) to ensure continuity of care. The challenge the inpatient team faced is the need to interface with community mental health teams from two London boroughs, as the unit became the main admission hospital for Kensington & Chelsea and Westminster (KCW) patients.

The main aim is that 80% of KCW patients' CMHH (including new referrals) will be contacted within 24 hours of them being admitted onto the ward by April 2024.

**Methods:**

As part of this QI project, weekly meetings were commenced, with a team comprising doctors, nursing staff (both inpatient and from local community team) and an Expert by Experience (EbE). A questionnaire was produced and circulated to ward colleagues about their views on the quality of whiteboard. A more focused questionnaire was then sent out around CMHH involvement in a patient's admission journey. We took a deep dive into the structure of the local community teams (at least 10 identified) and how referral processes work, as it was evident that staff were unclear at times on who/how to refer.

From this, the first change idea was formed: “information sheets” were produced showing which GPs correspond to which teams, and that patients can be referred this way. The Plan Do Study Act (PDSA) was applied to make these sheets visible to all staff. The outcome measure used was how many patients had CMHH referral/contact within 24 hours.

**Results:**

Data is being collected daily, by reviewing patients notes to see if CMHHs have been contacted. Since commencement of the first PDSA cycle in December 2023, of the twenty-three patients admitted, nineteen have been eligible. Of these nineteen patients, fifteen patients (79%) have had contact or referrals made to their CMHH within 24 hours.

**Conclusion:**

Results suggest that the aim is on the way to being met. Our next change idea is to obtain formal feedback from staff and patients on this process.

